# Glucose/ROS dual-responsive mitochondria-targeting nanozyme microneedles remodel mitochondrial homeostasis to orchestrate immune metabolic reprogramming for diabetic foot ulcer healing

**DOI:** 10.1093/rb/rbag153

**Published:** 2026-07-21

**Authors:** Qi Dong, Siyu Xiong, Bing Shu, Zhiwei Cai, Shangqing Wang, Yang Ming, Xiongwei Dong, Peng Chen, Zhaowei Zhang

**Affiliations:** School of Bioengineering and Health, Wuhan Textile University, Wuhan 430073, PR China; School of Bioengineering and Health, Wuhan Textile University, Wuhan 430073, PR China; Department of Neurosurgery, Zhongnan Hospital of Wuhan University, Wuhan 430060, PR China; School of Bioengineering and Health, Wuhan Textile University, Wuhan 430073, PR China; School of Bioengineering and Health, Wuhan Textile University, Wuhan 430073, PR China; Hubei Integrative Technology and Innovation Center for Advanced Fiberous Materials, Wuhan 430073, PR China; School of Bioengineering and Health, Wuhan Textile University, Wuhan 430073, PR China; Department of Pharmacy, Renmin Hospital of Wuhan University, Wuhan 430060, PR China; School of Bioengineering and Health, Wuhan Textile University, Wuhan 430073, PR China

**Keywords:** mitochondrial homeostasis, nanozymes, microneedle patches, immunometabolic reprogramming, diabetic wound healing

## Abstract

Diabetic foot ulcers (DFUs) develop a persistent vicious loop featuring impaired mitochondrial activity and massive buildup of reactive oxygen species (ROS), accompanied by disordered immune responses; these combined pathological changes hinder the regeneration of damaged tissues. Therapeutic strategies that spatiotemporally target mitochondria to couple redox homeostasis restoration, immunometabolic reprogramming and tissue regeneration remain an unmet clinical need. Here we report a glucose/ROS dual-responsive microneedle patch incorporating mitochondria-targeting metal–phenolic nanozymes for DFU treatment. Tannic acid-cerium (TA-Ce) nano-catalysts possess strong catalytic capacities analogous to natural superoxide dismutase and catalase enzymes. These dual biomimetic functions clear excess ROS, stabilize mitochondrial physiological balance and drive macrophages to shift from pro-inflammatory M1-type toward anti-inflammatory M2 subtypes, which ultimately breaks sustained inflammatory feedback loops. Notably, the nanozyme functions dually as a therapeutic agent and a dynamic crosslinker through reversible boronate ester chemistry, allowing stable loading and microenvironment-responsive, on-demand release aligned with the pathological features of diabetic wounds. In a full-thickness diabetic rat wound model, the microneedle patch achieves 92.64% wound closure on Day 14, significantly outperforming commercial dressings. This work establishes a mitochondria-centered immunometabolic therapeutic strategy and provides a translatable platform for chronic wound regeneration.

## Graphical abstract

**Graphical Abstract:**
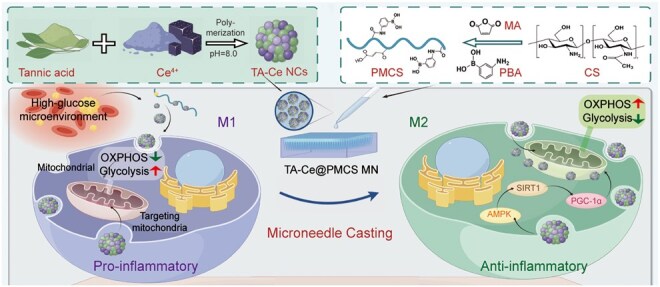

## Introduction

The clinical and socioeconomic burden of diabetic foot ulcers (DFUs) is staggering: in the USA alone, DFU management incurs annual healthcare costs exceeding $11 billion [1], and the condition is responsible for over 70 000 non-traumatic lower-limb amputations each year, which drastically reduce patient quality of life and increase 5-year mortality to over 50% [2, 3]. Current standard-of-care treatments for DFUs, including wound offloading, surgical debridement, systemic antibiotic therapy and basic moisture-retentive dressings, predominantly address symptomatic manifestations rather than the root pathological drivers of impaired healing [4–6]. These strategies exhibit limited therapeutic efficacy in clinical practice and are often accompanied by systemic side effects, high recurrence rates and an inability to reverse the chronic wound microenvironment [7]. There is thus an urgent and unmet clinical need to develop mechanism-driven, multifunctional therapeutic platforms that target the core pathophysiology of DFUs to restore physiological wound healing.

The impaired healing trajectory of DFUs is defined by a pathognomonic triad: persistent and non-resolving inflammation, impaired cellular proliferative and regenerative capacity, and dysregulated extracellular matrix (ECM) remodeling. At the heart of this pathological dysregulation lies a self-perpetuating vicious cycle between oxidative stress and aberrant inflammation, which is uniquely amplified under hyperglycemic conditions. Hyperglycemia disrupts systemic and local redox homeostasis, driving superoxide anion production at rates nearly 50-fold higher than physiological levels [8]. Excessive accumulation of reactive oxygen species (ROS) not only inflicts direct oxidative damage to nucleic acids, proteins and lipids but also triggers robust activation of pro-inflammatory signaling cascades, including the tumor necrosis factor-α (TNF-α) and interleukin-1β (IL-1β) pathways. Such biological reactions then recruit large quantities of neutrophils and pro-inflammatory M1-type macrophages, whose triggered nicotinamide adenine dinucleotide phosphate hydrogen oxidase-dependent respiratory burst reactions will worsen the overall oxidative stress status [9]. The resulting feedforward loop of ROS overproduction and chronic inflammation potently inhibits angiogenesis, re-epithelialization and ECM synthesis, creating a non-permissive microenvironment for wound healing that is refractory to conventional treatments [10]. While numerous antioxidant and anti-inflammatory strategies have been developed to break this cycle, most fail to achieve durable therapeutic benefits in DFUs, largely due to their inability to address the upstream cellular driver of both ROS overproduction and inflammatory dysregulation.

Emerging evidence has established mitochondrial metabolism as the central regulatory hub governing macrophage phenotypic polarization and functional fate, which is the critical determinant of inflammation resolution and wound repair. Classically activated M1 macrophages obtain energy mainly through aerobic glycolysis, accompanied by impaired tricarboxylic acid cycle and weakened mitochondrial oxidative phosphorylation (OXPHOS). In contrast, reparative M2 macrophages maintain their anti-inflammatory and wound-repair bioactivities by relying on stable mitochondrial OXPHOS and fatty acid metabolic pathways [11]. Notably, the hyperglycemic microenvironment in DFUs directly induces profound mitochondrial dysfunction, including impaired respiratory chain complex activity, loss of mitochondrial membrane potential (MMP), reduced mitochondrial biogenesis and excessive mitochondrial ROS production [12–14]. This mitochondrial dysfunction is not only the primary source of pathological ROS overproduction in DFUs but also locks macrophages in a pro-inflammatory glycolytic M1 phenotype, further amplifying the ROS-inflammation vicious cycle and stalling wound healing [15, 16]. Therefore, therapeutic strategies that can simultaneously restore mitochondrial homeostasis, normalize macrophage immunometabolic phenotype and break the ROS-inflammatory cycle represent a transformative paradigm shift in DFU treatment. However, to date, there are few translatable therapeutic platforms that can achieve spatiotemporally controlled, mitochondria-targeted delivery of therapeutics to the DFU lesion while coupling mitochondrial homeostasis restoration with immunometabolic reprogramming and tissue regeneration.

Metal-phenolic networks (MPNs), constructed via the coordination of natural polyphenols with diverse metal ions, have emerged as a versatile class of functional nanomaterials for biomedical applications, owing to their facile synthesis, tunable catalytic activity, intrinsic bioactivity and substantial biocompatibility [17, 18]. Unlike monofunctional inorganic nanozymes (e.g. CeO_2_, Mn_3_O_4_, V_2_O_5_), MPN-based nanozymes integrate the inherent biological activities of polyphenols with the robust enzyme-mimetic catalytic properties of metal ions, enabling multifunctional therapeutic performance with a single nanostructure [19–21]. For instance, curcumin-iron (Cur-Fe) MPNs nanozymes achieve concurrent ROS scavenging and inflammatory modulation for acute lung injury treatment [22, 23], while tannic acid-copper (TA-Cu) MPNs nanozymes enable antibacterial, antioxidative and osteogenic activities for periodontal disease management [24]. TA, a natural plant-derived polyphenol, is an ideal building block for MPNs nanozymes: it exhibits inherent mitochondria-targeting capability via hydrogen bonding, hydrophobic interactions and π–π stacking with mitochondrial outer membrane proteins, as well as potent intrinsic antioxidant, anti-inflammatory and mitochondrial protective activities. However, the therapeutic efficacy of free TA is severely limited by its low bioavailability, poor chemical stability and lack of spatiotemporal control over release *in vivo*. Cerium-based nanoparticles exert broad-spectrum and durable antioxidant activity via the reversible Ce(III)/Ce(IV) redox cycle, which confers dual superoxide dismutase (SOD)- and catalase (CAT)-mimetic activities to eliminate multiple ROS species [25, 26]. On this basis, we rationally designed a TA-Ce MPN nanozyme, which integrates the mitochondria-targeting and mitochondrial protective properties of TA with the dual enzyme-mimetic ROS-scavenging activity of Ce(III)/Ce(IV) redox pairs. We hypothesized that this TA-Ce nanozyme could target mitochondria to eliminate pathological ROS, restore mitochondrial structure and function, and thereby drive immunometabolic reprogramming of macrophages to resolve chronic inflammation in DFUs.

Despite the therapeutic promise of antioxidant nanozymes, their clinical translation for DFU treatment is hindered by two critical technical challenges. First, the intact skin stratum corneum presents a major barrier to transdermal delivery of nano-formulations, making it difficult to achieve sufficient and sustained drug accumulation in deep wound tissues via topical administration. Second, conventional drug delivery systems exhibit uncontrolled burst release, which mismatches the dynamic progression of the DFU microenvironment, leading to insufficient therapeutic efficacy at the pathological site and potential off-target side effects [16]. Stimuli-responsive “smart” hydrogels, which can undergo adaptive structural and physicochemical changes in response to pathological cues in the wound microenvironment (e.g. pH, ROS, glucose, temperature), offer a promising solution to achieve on-demand, spatiotemporally controlled drug release [27, 28]. When integrated with microneedle patch (MN) technology, these smart hydrogels can overcome the skin barrier to enable minimally invasive, localized, and precise transdermal delivery of therapeutics directly into the wound bed, further enhancing therapeutic efficacy while minimizing systemic exposure. However, most existing smart microneedle systems rely on simple physical encapsulation of therapeutic agents, which often leads to premature drug leakage, impaired bioactivity of the payload, and insufficient responsiveness to the DFU microenvironment. There remains a critical need to develop smart microneedle platforms where the therapeutic nanozyme itself serves as an active crosslinker to form the dynamic hydrogel network, enabling highly stable payload loading, microenvironment-specific responsive release, and full retention of therapeutic bioactivity.

Herein, we report a novel, microenvironment-adaptive smart hydrogel microneedle platform based on TA-Ce MPNs nanozymes, engineered to reprogram the DFU microenvironment via mitochondria-centric immunometabolism regulation. In this design, TA-Ce nanozymes with mitochondria-targeting capability, dual SOD/CAT-mimetic activities and intrinsic anti-inflammatory properties serve as both the bioactive therapeutic payload and the multi-site dynamic crosslinker, forming a reversible hydrogel network with phenylboronic acid (PBA)-functionalized methylacrylate chitosan (PMCS) via boronate ester bonds between the catechol groups of TA and the PBA moieties of PMCS. The resulting dynamic hydrogel was further fabricated into an MN (TA-Ce@PMCS MN) via a facile mold-casting method. The core objectives and innovations of this study are as follows: (i) We developed a TA-Ce MPN nanozyme with potent broad-spectrum ROS-scavenging activity and intrinsic mitochondria-targeting capability, which can restore mitochondrial homeostasis and drive M1-to-M2 macrophage phenotypic transition via immunometabolic reprogramming, thereby breaking the core ROS-inflammation vicious cycle in DFUs. (ii) We engineered a glucose/ROS dual-responsive microneedle platform, where the TA-Ce nanozyme acts as a dynamic crosslinker to enable highly stable loading and on-demand, microenvironment-triggered release of the nanozyme specifically in hyperglycemic, high-ROS DFU lesions, achieving spatiotemporally controlled delivery aligned with wound healing dynamics. (iii) We systematically demonstrated that TA-Ce@PMCS MN significantly accelerates diabetic wound healing *in vivo* via concurrent redox balance restoration, macrophage immune reprogramming, enhanced angiogenesis and promoted collagen deposition. This work not only establishes a facile and translatable mitochondria-centric immunometabolic regulatory platform for DFU treatment but also provides a universal design paradigm for engineering MPN-based smart microneedle systems for the management of chronic inflammatory diseases and tissue regeneration disorders.

## Experimental section

### Materials

Chitosan (CS) was obtained from Qingdao ChiBio Biotech Co., Ltd., China. The apparent viscosity of CS was 1000 mPa·s. The degree of deacetylation was determined to be 95.2%. The measured weight-average molecular weight (Mw) was 3.2 × 10^5^ Da, the number-average molecular weight (Mn) was 1.8 × 10^5^ Da, and the polydispersity index (Mw/Mn) was 1.78. TA, Ce (SO_4_)_2_, lithium phenyl(2,4,6-trimethylbenzoyl) phosphate (LAP), and NaOH were purchased from Shanghai Aladdin Biochemical Technology Co., Ltd. Maleic anhydride, 3-aminophenylboric acid, N-hydroxysuccinimide (NHS) and 1-ethyl-3-(3-dimethylaminopropyl) carbodiimide hydrochloride (EDC·HCl) were obtained from Sinopharm Chemical Reagent Co., Ltd. All other reagents were A.R. grade. SIRT1 (1:5000, Abcam ab110304), PGC-1α (1:1000, Abcam ab54481), p-AMPK (1:2000, HUABIO, HA723603), AMPK (1:2000, HUABIO, HA723038, 5832) were obtained from Abcam.

### Synthesis and characterization of maleimide-PMCS

Synthesis of PMCS was completed with two separate coupling reaction stages. The synthesis reaction proceeded by dissolving 1 g CS powder and 3.5 g maleic anhydride within 100 mL DMSO solvent. The mixed solution was incubated at a constant temperature of 55°C for 24 h. We adjusted the pH of the reaction liquid using sodium bicarbonate, followed by product precipitation in acetone solvent. Solid sediments were harvested via filtration, followed by thorough dialysis in deionized water. Subsequent freeze-drying treatment finally yielded the target maleimide-modified chitosan product (MCS). For the next synthetic step, 0.5 g of MCS was mixed with 0.32 g NHS and 0.5 g EDC·HCl in 100 mL of pH 5.0 PBS buffer. The mixture underwent a 1-h activation process. After activation, 0.5 g of 3-aminophenylboronic acid was introduced into the reaction system, and the mixture was continuously reacted for 24 h. Post-reaction centrifugation was performed to separate the solution components, and the collected supernatant was further dialyzed and freeze-dried to acquire the final PMCS product. The chemical structures of MCS and PMCS were verified by ^1^H NMR (Bruker AV 400 NMR, USA) with D_2_O/CD_3_COOD as the solvent. Functional group characterization of the prepared samples was conducted utilizing a Nicolet-8700 FTIR spectrometer from Thermo USA. All spectroscopic tests were implemented at ambient temperature, with the scanning wavenumber range set from 4000 to 400 cm^−1^.

### Synthesis and characterization of TA-Ce nanozyme

The TA-Ce nanozyme was synthesized via a self-assembly approach. Briefly, TA (2 g) was dissolved in aqueous Ce (SO_4_)_2_ solution (170 mL, 24 mM), and the pH was adjusted to 8.0 using 1 M NaOH. Following a 15-min reaction period, the synthesized TA-Ce nanoparticles were harvested through centrifugation and rinsed thoroughly with deionized water in three repeated washes. Transmission electron microscopy (TEM) equipment manufactured by Thermo Fisher was applied to observe particle microscopic morphology, while integrated EDS mapping technology enabled intuitive analysis of elemental distribution. A Malvern Nano-ZS90 instrument was employed for dynamic light scattering detection, which acquired the hydrodynamic particle size and surface zeta potential of the prepared nanoparticles. The TA-Ce coordination was verified by Fourier-transform infrared spectroscopy (FTIR; Nicolet iS50, Thermo Scientific, USA). Cerium ionic states (Ce³^+^/Ce^4+^ ratio) were determined by X-ray photoelectron spectroscopy (XPS; Thermo Fisher Scientific, USA). TA content in TA-Ce nanozymes was quantified by thermogravimetric analysis (Smart-Seal Pan, USA).

### *In vitro* characterization of ROS scavenging capability

SOD-like activity of tannic cerium nanoparticles was quantified using a commercial SOD assay kit (Solarbio). Following the operating guidelines attached to the detection kit, gradient TA-Ce nanoparticle samples were mixed with superoxide anion testing liquid and kept in a 37°C environment for 40 min. After another 10-min reaction with color-developing agent, optical density values at 550 nm were recorded to calculate the free radical clearance capacity.

CAT-like activity was evaluated via a hydrogen peroxide assay kit (Solarbio). Catalytic H_2_O_2_ consumption (conversion to H_2_O and O_2_) was monitored by measuring residual H_2_O_2_ concentration.

The hydroxyl radical scavenging ability of the samples was determined using the salicylic acid-Fe^2+^-H_2_O_2_ reaction system. •OH radicals were generated by reacting salicylic acid (9 mM) and FeSO_4_ (9 mM) with H_2_O_2_ (8.8 mM). Diverse dosages of TA-Ce nanomaterials underwent interactions with hydroxyl radical detection liquid at 37°C for half an hour. Recorded optical density readings at 430 nm were utilized to quantify the sample’s SOD-like activity.

The ROS and RNS scavenging ability of TA-Ce nanozyme was assayed via 1,1-diphenyl-2-picrylhydrazyl (DPPH) and 2-phenyl-4,4,5,5-tetramethylimidazoline-3-oxide-1-oxyl (PTIO), respectively. DPPH solution (100 µM) was reacted with gradient concentrations of TA-Ce, and after reaction in the dark for 30 min, the absorbance peak at 517 nm was measured. For the PTIO assay, PTIO (1.5 wt%) was co-cultured with nanozymes with gradient concentrations for 2 h. Scavenging efficiency was quantified by absorbance reduction at 557 nm.

### Synthesis of PMCS and TA-Ce@PMCS hydrogel

To prepare TA-Ce@PMCS hydrogel, PMCS (1 w/w%) and TA-Ce (1 mg/mL) were dissolved in deionized water (10 mL). The LAP photoinitiator was incorporated at a proportion of 0.1 w/w%, followed by 1 h of continuous stirring to achieve uniform dispersion. The homogeneous precursor solution was then subjected to 365 nm Ultraviolet (UV) irradiation with an optical power density of 30 mW/cm^2^ for 60 s, which triggered crosslinking and successfully formed the target TA-Ce@PMCS hydrogel. For control group preparation, pure PMCS hydrogel was fabricated through identical reaction conditions without the addition of TA-Ce nanoparticles. Microstructural features of freeze-dried hydrogel samples were visualized via scanning electron microscopy. Combined XPS and FTIR characterization further enabled comprehensive analysis of the material’s chemical composition and structural properties. Gelation kinetics were monitored using a MARS III rheometer (Thermo Fisher, Germany) at 25°C under UV illumination (30 mW/cm^2^). Rheological characterization included amplitude sweeps (0.1–10000% strain, 1 Hz) to determine fracture points; frequency sweeps (0.1–10 Hz, 1% strain); and dynamic step-strain measurements (alternating 1% and 1000% strain, 1 Hz, 60-s intervals) tracking storage (G′) and loss (G″) moduli. The ROS/glucose dual-responsive property and swelling behavior of the hydrogels were tested by exposing the samples to 1 mM H_2_O_2_ and 25 mM glucose. The crossover of storage modulus (G′) and loss modulus (G″) was monitored by rheometry.

### Fabrication and characterization of TA-Ce@PMCS MNs

Bilayer TA-Ce@PMCS MN patches were fabricated via a two-step mold-casting protocol using laser-etched polydimethylsiloxane (PDMS) templates (20 × 20 array; needle height = 800 µm). Briefly, 1 mL of the TA-Ce@PMCS hydrogel precursor was loaded into the PDMS cavity and driven into the conical cavities by sequential vacuum de-bubbling (–0.1 MPa, 5 min) and solvent evaporation cycles. The needle tips were subsequently cross-linked under 365 nm UV irradiation (60 mW/cm^2^, 60 s). A 20 wt% poly (vinyl alcohol) backing solution (1 mL) was then cast onto the solidified tips and dried at ambient conditions for 24 h to yield a mechanically robust base layer. Once fully dried, the fabricated MNs were carefully demolded from PDMS templates and preserved inside a desiccator prior to subsequent experiments. A Hitachi Regulus8100 field-emission scanning electron microscope was utilized to capture high-resolution images of microneedle topography and interfacial structures. Meanwhile, axial compression tests were performed to quantitatively evaluate the mechanical performance and structural stability of the prepared microneedle samples. We adopted an Instron 5943 material tester fitted with a 500 N force sensor, setting the compression rate to 0.1 mm per minute for all tested samples. TA-Ce release profiles were monitored in triplicate (*n* = 3) under physiologically relevant oxidative–hyperglycemic conditions (1 mM H_2_O_2_, 25 mM glucose, 37°C). One milliliter of the hydrogel samples was placed in 5 mL of PBS (containing 1 mM H_2_O_2_ and 25 mM glucose). At every scheduled detection time, we retrieved the PBS solution and replaced it with fresh buffer liquid. Three parallel replicates were prepared for every experimental group (*n* = 3).

### Cytocompatibility analysis

Cytocompatibility of the TA-Ce@PMCS MN patches was evaluated on L929 murine fibroblasts by MTT reduction and live/dead dual-fluorescence staining. Extracts were prepared by immersing sterilized MN patches in serum-free Dulbecco’s Modified Eagle Medium (DMEM) (0.2 g/mL) for 72 h at 37°C. L929 cells were seeded in 96-well plates (1 × 10³ cells per well), and after 24-h equilibration, the medium was exchanged with 100-µL extract. Metabolic activity was quantified on Days 1 and 2 using the MTT assay; formazan absorbance was recorded at 490 nm (Multiskan FC, Thermo Scientific). To observe cellular morphological features, cells received a 30-min staining treatment using calcein-AM and propidium iodide (PI) at 37°C, followed by image capture via an inverted fluorescence microscopic device.

### Intracellular antioxidant evaluation

Intracellular ROS levels in RAW264.7 macrophages were quantified using the cell-permeable fluorogenic probe 2′,7′-dichlorofluorescein diacetate (DCFH-DA). Cells were seeded at 5 × 10^4^ cells per well in six-well plates and allowed to adhere for 12 h. The culture medium was then replaced with serum-free DMEM containing 200 µM H_2_O_2_ and incubated for 6 h to induce oxidative stress. Subsequently, the medium was exchanged with fresh serum-free DMEM supplemented with TA-Ce@PMCS MNs for an additional 12 h. Following the experimental intervention, cellular samples underwent two PBS rinses. They were then cultured with 10 µM DCFH-DA solution at 37°C for 20 min under light-shielded conditions. Following three additional PBS rinses, intracellular ROS-associated fluorescence was captured by laser-scanning confocal microscopy (LSCM; Leica TCS SP8).

### Anti-inflammatory activity

RAW264.7 macrophages (5 × 10^5^ cells per well) were seeded in six-well plates for 24 h, then stimulated with 100 ng/mL LPS for 12 h to induce the pro-inflammatory (M1) phenotype. LPS-containing medium was replaced with fresh medium supplemented with TA-Ce@PMCS MN extracts. After 12-h co-culture, cells were fixed (4% paraformaldehyde, 15 min), permeabilized (0.1% Triton X-100) and sequentially incubated with primary antibodies against CD86 (M1) and CD206 (M2) (4°C, 12 h) and Alexa Fluor-conjugated secondary antibodies (30 min). Nuclei were counterstained with DAPI. Fluorescence intensities were analyzed by LSCM and ImageJ. For gene-expression profiling, total RNA was extracted using Trizol, reverse-transcribed to cDNA, and analyzed by reverse transcription quantitative polymerase chain reaction (RT-qPCR) for CCR7, IL-1β, TNF-α, IL-6, ARG-1, CD163, IL-10 and TGF-β. Primer sequences are provided in Supplementary Table S2.

### *In vivo* diabetic wound-healing assay

We built an *in vivo* diabetic cutaneous wound model based on 8-week-old female Sprague–Dawley rats weighing between 220 and 250 g. Wuhan Myhalic Biotechnological Co., Ltd. provided all experimental rodents, and every animal operation strictly complied with standards stated in the National Research Council’s laboratory animal care manual (approval number HLK-20250617-001). The diabetic rats were induced by injecting intraperitoneally STZ (60 mg/kg). Seven days after completing the injection operation, we drew blood samples from the rat tail veins to test glucose concentrations. Animals whose casual blood glucose exceeded 16.7 mmol/L were regarded as qualified diabetic models. We separated 30 eligible diabetic rodents into three test batches at random, including standard 3M Tegaderm™ film, blank PMCS microneedles, and TA-Ce@PMCS microneedles, with 10 individuals allocated to each group. Each rat received anesthetic treatment via 1% (w/v) sodium pentobarbital, and then two round, full-thickness skin defects measuring 15 mm across were generated on the animal’s back area. MNs were applied, and wound closure was documented by digital photography on Days 0, 3, 7, 10 and 14.

### Histology and immunofluorescence analysis

Tissue specimens taken from wound regeneration sites on Days 7 and 14 were preserved in 4% paraformaldehyde solution, embedded in paraffin wax and sliced into 4-µm-thin sections. We conducted Hematoxylin–eosin (H&E) staining to assess epithelial regrowth and Masson’s trichrome staining for collagen accumulation analysis; ImageJ software was utilized to calculate epidermal thickness and collagen density values.

For immunofluorescence, Day 7 sections were incubated with DCFH-DA to visualize ROS; Day 7 sections were probed with primary antibodies against CD86 and CD206, followed by fluorophore-conjugated secondary antibodies. Nuclei were labeled with DAPI. On Day 14, neovascularization was assessed by CD31 and vascular endothelial growth factor (VEGF).

### Statistical analysis

All quantitative results were expressed as mean values with standard deviation (SD). Origin 8.0 and ImageJ software handled all statistical calculations. Single-factor analysis of variance combined with Tukey post hoc comparison was adopted to identify intergroup disparities. Markers of statistical significance were defined as *P < 0.05, **P < 0.01 and ***P < 0.001, respectively.

## Results and discussion

### Fabrication and characterization of TA-Ce nanozyme

**Figure 1:**
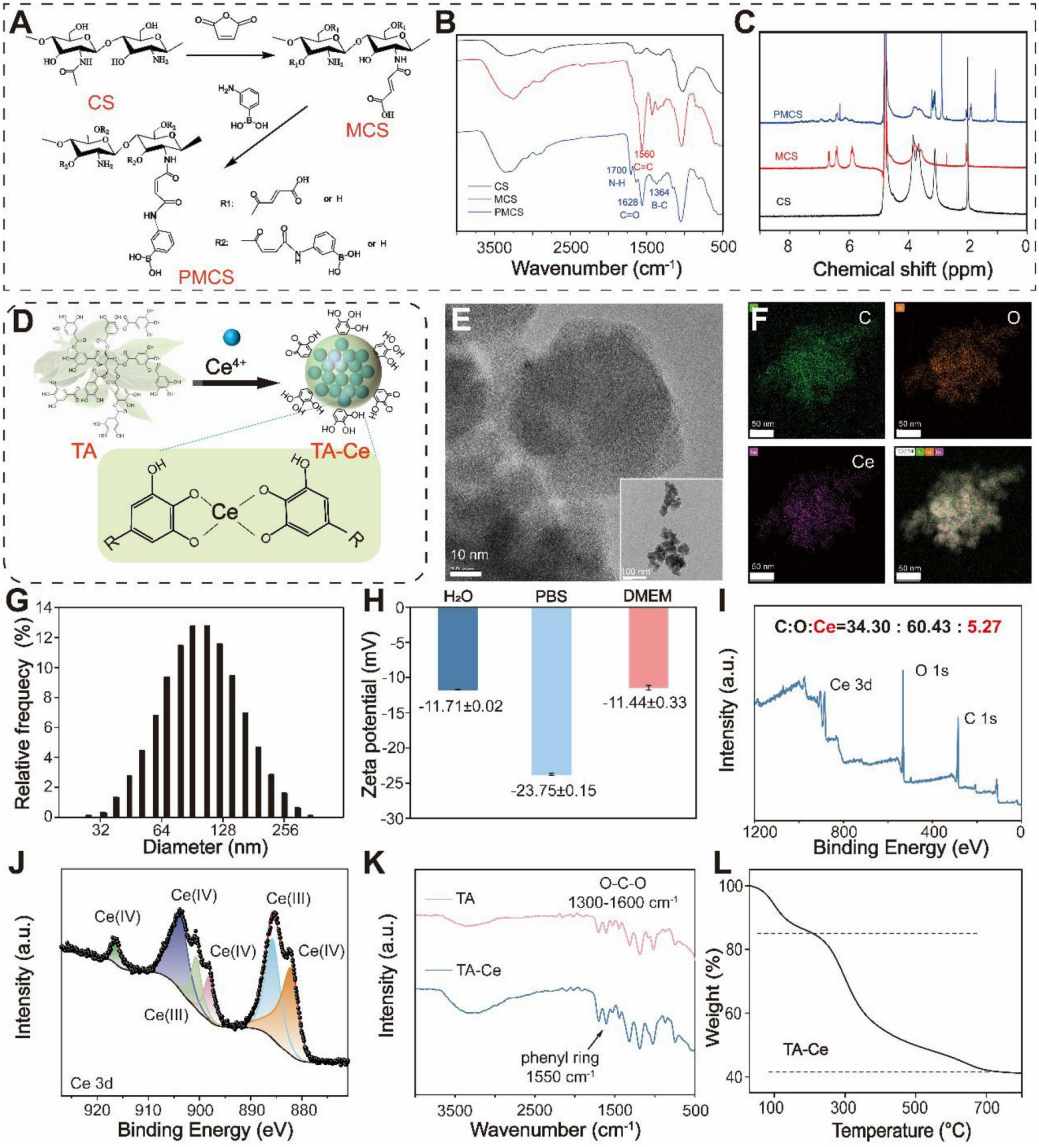
Synthesis and characterization of PMCS and TA-Ce nanozyme. (**A**) Schematic illustration of PMCS synthesis. (**B**) The FTIR spectra of CS, MCS and PMCS. (**C**) The ^1^H NMR spectra of CS, MCS and PMCS. (**D**) Schematic illustration of TA-Ce nanozyme synthesis. (**E**) TEM images of the TA-Ce nanozyme. (**F**) EDS elemental mapping images. (**G**) The dynamic light scattering (DLS) result of TA-Ce nanozyme. (**H**) Zeta potentials of the TA-Ce nanozyme in different solvents. (**I**) XPS profiles. (**J**) High-resolution XPS patterns of the Ce 3d peaks. (**K**) FTIR patterns of TA and TA-Ce nanozymes. (**L**) Thermogravimetric curves.

In this study, the PMCS was prepared by grafting maleimide and PBA onto CS (Figure 1A). The structure was characterized by ^1^H NMR and FTIR (Figure 1B and C). Following maleimide graft modification, characteristic peaks appeared at 3074 and 1560 cm^−1^ within FTIR spectra collected from MCS and PMCS samples. These two distinct signals separately correspond to vinyl C–H stretching vibrations and the olefinic double bond originating from attached maleoyl moieties. The absorption band at 1364 cm^−1^ in the conjugate represents the B(OH)_2_ stretching of PBA, and the absorption bands at 1628 and 1700 cm^−1^ represent C=O stretch and N–H bend, respectively, attributed to the formation of the amide bond in the conjugate [29, 30]. Further ^1^H NMR analysis showed several new peaks at 6.6, 6.3 and 5.8 ppm in MCS and PMCS compared with CS, which were attributed to the two protons of vinyl protons, and 7.5 ppm was attributed to the typical phenyl peak of PBA [31, 32]. The grafting ratios of C=C and PBA were 1.67 and 0.25, respectively. These results indicate the successful production of PMCS.

TA-Ce nanoparticles were synthesized via a one-step redox reaction approach, as shown in Figure 1D. TA was selected because phenolic groups can serve as binding sites to chelate and reduce Ce^4+^ under alkaline conditions [33]. As shown in Figure 1E and F, TA-Ce showed a nanoparticulate morphology with a diameter of approximately 50 nm and evenly distributed C, O, and Ce elements, with atomic fractions of Ce (2.75%), C (72.55%) and O (24.70%) (Supplementary Figure S1 and Table S1). Quantitative testing confirmed that cerium accounts for 23.33% of the total mass. Such results confirm that cerium components are evenly distributed within the inner structure of the prepared nanoparticles. Figure 1G and H displays the detected particle hydrodynamic sizes and surface zeta potentials. DLS detection data demonstrated that TA-Ce nanoparticles possessed an average hydrodynamic dimension of 95 nm. Zeta potential detection was further performed in three common experimental media, yielding values of −11.71 ± 0.02 mV in pure water, −23.75 ± 0.15 mV in PBS buffer and −11.44 ± 0.33 mV in DMEM medium. These measured values prove the favorable physiological stability of TA-Ce nanoparticles in such biological environments. The catalytic activity of TA-Ce nanozyme originates from the Ce³^+^/Ce^4+^ redox cycle. For CAT-like activity, H_2_O_2_ is decomposed into H_2_O and O_2_, and a higher Ce³^+^/Ce^4+^ ratio enhances the SOD-like activity. CAT-like activity relies more on Ce^4+^ [29, 34]. XPS was adopted to characterize TA-Ce nanoparticles, with a focus on analyzing surface elemental makeup and cerium valence distributions. As illustrated in Figure 1I and J, XPS characterization confirms the elemental makeup and successful synthesis of TA-Ce nanoparticles. Three obvious characteristic signals appear at 900, 533 and 285 eV, separately matching the Ce 3d, O 1s and C 1s orbital peaks. Both Ce³^+^ and Ce^4+^ oxidation states can be detected within the spectral profiles. According to previous research [35, 36], signals at 882.27, 901 and 916.06 eV originate from Ce^4+^, and the peaks at 886.09 and 904.71 eV correspond to Ce³^+^ ions. Quantitative analysis calculated the proportion of Ce^4+^ to reach 60.33%, indicating that the antioxidant tetravalent cerium constitutes the dominant valence state. High-resolution XPS spectral scanning targeting the C 1s orbit identified three characteristic peaks belonging to the synthetic TA-Ce sample. The dominant signal observed at 284.8 eV corresponds to carbon–carbon single bonds present in the aromatic molecular skeleton. A minor shoulder signal at 286.8 eV corresponds to carbon–oxygen single and double bonds. Additionally, the wide signal at 290.9 eV represents the π–π* shake-up satellite peak, a typical feature of surface aromatic rings on TA-Ce samples, as documented in prior literature [29] (Supplementary Figure S2). FTIR spectroscopic testing was utilized to verify the successful fabrication of TA-Ce nanoparticles (Figure 1K). Characteristic benzene ring vibration and substitution signals were detected around 1550 and 790 cm^−1^. Other distinct absorption bands were also observed across the scanning range. The spectral signals ranging from 1300 to 1600 cm^−1^, alongside peaks at 1700 and 3600 cm^−1^, are attributed to O–C–O, –C=O and –O–H functional groups in sequence. These typical functional group features fully validate the successful preparation of target TA-Ce materials, consistent with previous studies [26, 37]. Further thermogravimetric results of TA-Ce indicate that the assembled TA accounts for 42% of the total weight (Figure 1L). These results indicate that the TA-Ce nanozyme with potential antioxidant ability was successfully prepared.

### ROS scavenging activity of TA-Ce nanozyme

**Figure 2:**
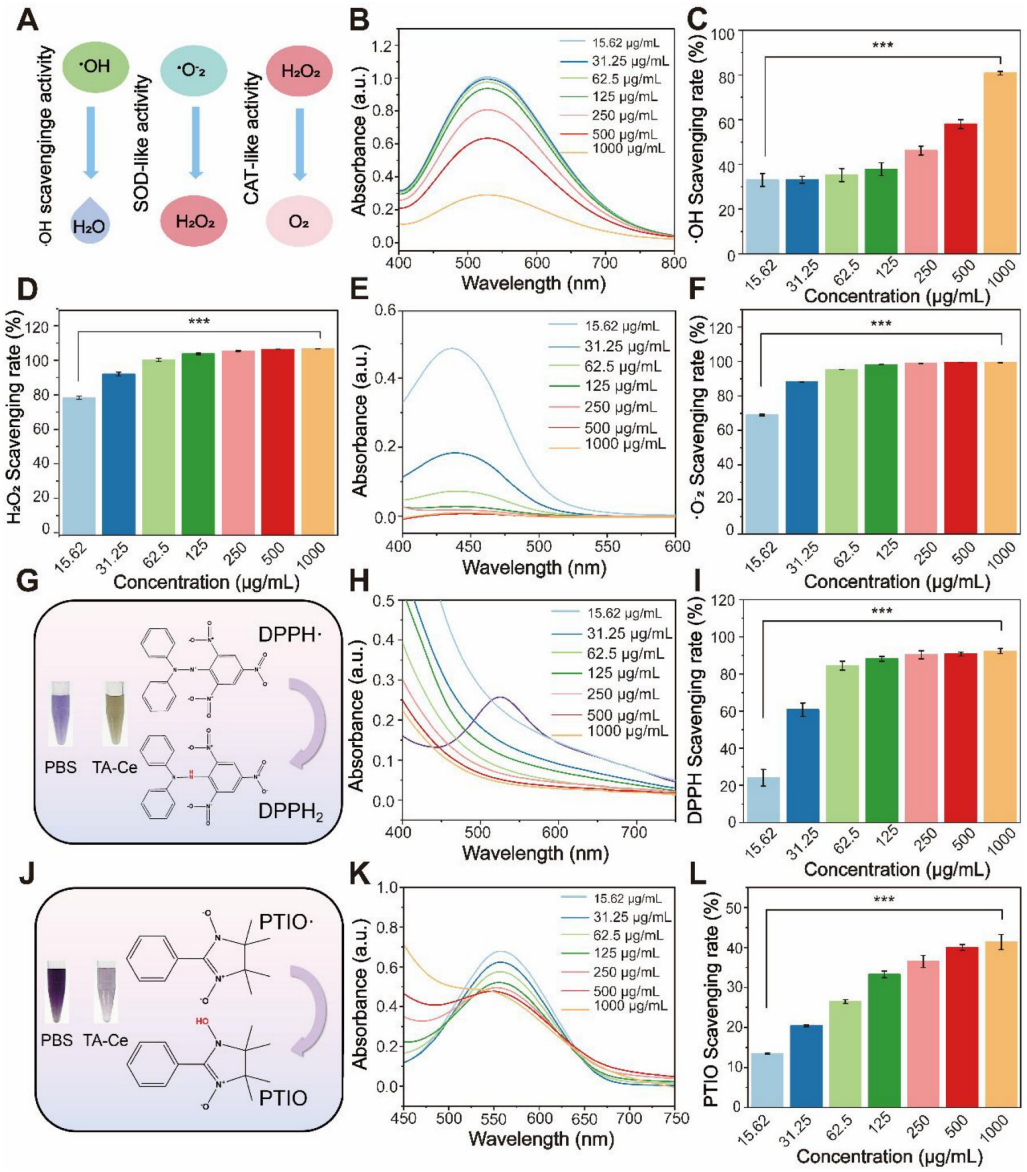
Characterization of the antioxidative performance of TA-Ce nanozyme. (**A**) Schematic illustration of the antioxidation mechanism of the TA-Ce nanozyme. (**B**) UV–Vis spectra of •OH removal with different concentrations of TA-Ce nanozyme. (**C**) •OH scavenging rate. (**D**) The H_2_O_2_ scavenging rate. (**E**) UV–Vis spectra of •O_2_^−^ removed with different concentrations of TA-Ce nanozyme. (**F**) •O_2_^−^ scavenging rate. (**G**) DPPH• clearance mechanism of the TA-Ce nanozyme. (**H**) UV–Vis spectra of DPPH• scavenging at different concentrations of TA-Ce nanozyme. (**I**) DPPH• scavenging rate. (**J**) Scavenging mechanism of the TA-Ce nanozyme for PTIO•. (**K**) UV–Vis spectra of PTIO• scavenging at different concentrations of TA-Ce nanozyme. (**L**) PTIO• scavenging rate (*n* = 3, ***P < 0.001).

**Figure 3:**
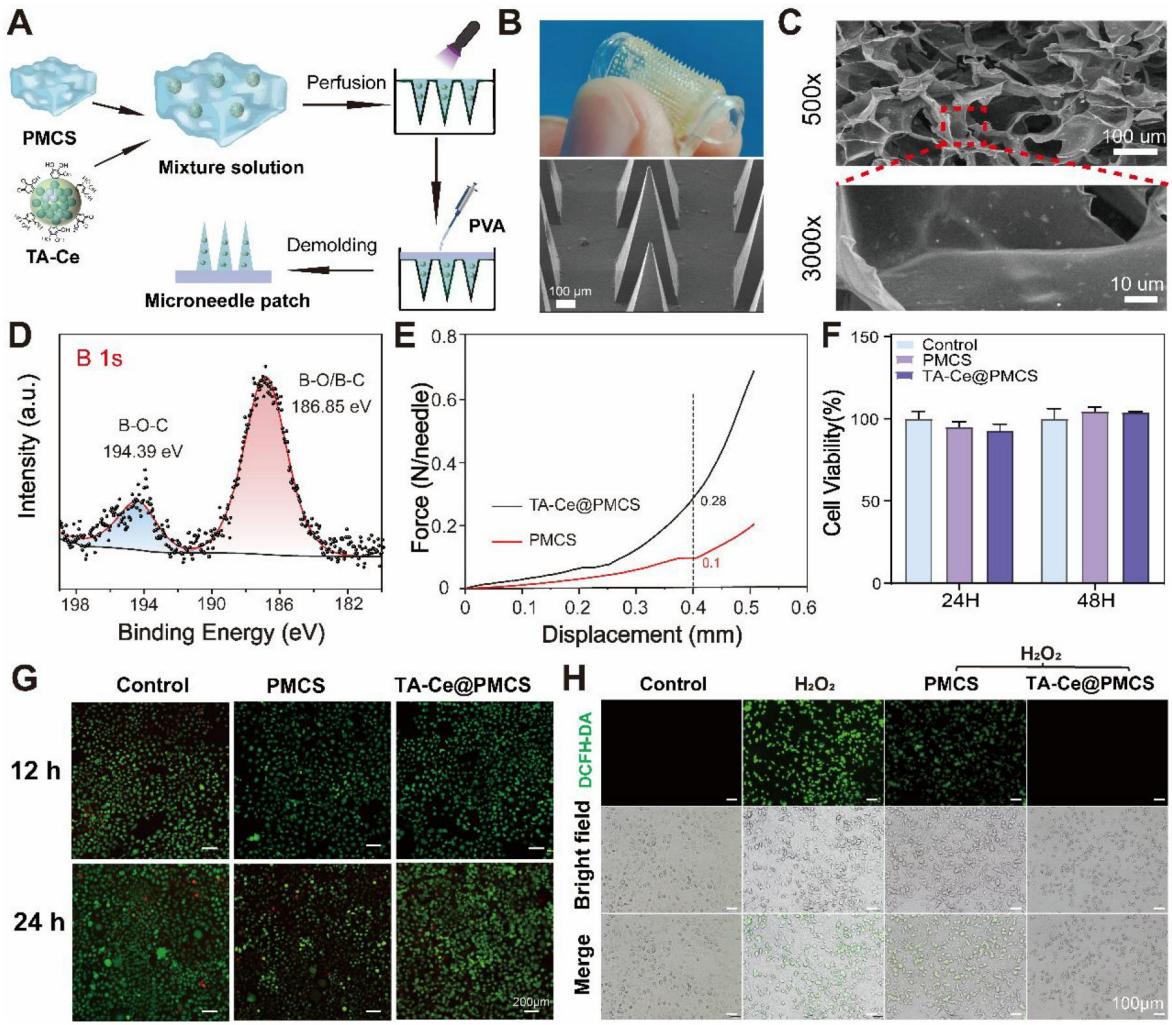
Characterization of TA-Ce@PMCS MN. (**A**) Schematic diagram of the synthesis of TA-Ce@PMCS MN. (**B**) Phototropic and SEM images of the MN. (**C**) The microstructure of tips. (**D**) B 1s XPS spectra. (**E**) Force–displacement curves of MN. (**F**) The cell viability of L929 cells after co-culturing for 48 h. (**G**) Calcein-AM/PI staining outcomes following a 24-h incubation. (**H**) CLSM images of intracellular ROS levels using by DCF-DA fluorescence probe.

The most obvious feature of DFU is a high level of ROS, which can seriously destroy the structure and function of intracellular macromolecules [38, 39]. We assessed the ROS scavenging capability of TA-Ce nanozymes from multiple antioxidant dimensions (Figure 2A). The assessment system covered catalytic breakdown of hydrogen peroxide, SOD-mediated superoxide anion clearance, hydroxyl radical removal efficiency and overall antioxidant level. As a highly harmful ROS, hydroxyl radicals were produced via a classic Fenton reaction model. In this experimental setup, ferrous ions and hydrogen peroxide served as core reactants to generate •OH free radicals [40]. Salicylic acid can react with hydroxyl radicals to generate specific derivatives with a characteristic absorption peak at 510 nm. According to the testing results in Figure 2B, elevated TA-Ce concentrations effectively lowered the absorbance intensity at this specific wavelength. Notably, the hydroxyl radical elimination rate rose markedly with higher TA-Ce dosages. The scavenging efficiency reached 81% at the concentration of 1000 µg/mL, a sharp improvement from the 33% recorded at 15.62 µg/mL, which validates the substantial •OH removal capacity of TA-Ce nanozymes (Figure 2C). The CAT-like catalytic performance was evaluated by detecting variations in hydrogen peroxide levels. Superoxide disproportionation reactions continuously produce H_2_O_2_, and the synthesized nanozymes can break down such accumulated hydrogen peroxide into harmless water and oxygen molecules [41, 42]. As shown in Figure 3D, TA-Ce nanozyme exhibits efficient hydrogen peroxide decomposition, with about a 100% scavenging ratio at 125 µg/mL. The SOD-mimic property was further tested using an SOD assay kit. As shown in Figure 3E and F, the product-soluble methane of •O_2_^−^ and tetrazolium salt shows an obvious absorption. With the introduction of TA-Ce nanozyme, the absorption peak decreased significantly; the •O_2_^−^ scavenging ratio of TA-Ce is 69% at 15.62 µg/mL, increased to 99% at 62.5 µg/mL, indicating that TA-Ce nanozyme is highly effective in removing the most toxic superoxide anions. Collectively, the synthesized TA-Ce nanozyme achieves substantial scavenging effects on diverse ROS. It mimics the synergistic catalytic cascade of SOD and CAT enzymes, which first facilitates the dismutation of superoxide anions into hydrogen peroxide and further transforms the generated H_2_O_2_ into oxygen and water molecules [43].

To further explore the free radical elimination performance of the material, two common radical probes were adopted for functional evaluation. DPPH serves as a classic nitrogen-centered radical, while PTIO represents typical oxygen-centered radicals, both applied to test the free radical scavenging efficiency of the prepared samples [44]. Upon mixing DPPH reagents with TA-Ce nanozymes, the optical absorption intensity decreased distinctly along with gradual solution decolorization, which strongly proves the material’s outstanding DPPH radical clearance capacity (Figure 2G). Corresponding quantitative data displayed in Figure 2H and I demonstrate that TA-Ce delivers dose-dependent radical scavenging performance. Within the concentration range from 15.62 to 1000 µg/mL, the 30-min DPPH removal rate increased from 24% up to 90%. Meanwhile, the PTIO scavenging rate reached 42% after 2 h of incubation, starting from an initial 13.5% (Figure 2J–L). Overall, TA-Ce possesses robust antioxidant properties that can eliminate multiple ROS such as H_2_O_2_, •OH and •O_2_^−^. Such substantial performance enables TA-Ce to relieve intracellular oxidative damage and protect cells and tissues from oxidative stress. Furthermore, the long-term stability of the TA-Ce nanozyme was analyzed by immersing the TA-Ce nanozyme in PBS for 14 days. As shown in Supplementary Figure S3, the particle size remains highly stable over time (Day 0–14), with no statistically significant changes, indicating substantial structural stability. Meanwhile, the DPPH radical scavenging activity shows only a slight decrease from 92.31% at Day 0 to 85.32% at Day 14, suggesting that the nanozyme retains most of its antioxidant capacity after prolonged incubation.

These results collectively demonstrate that TA-Ce nanozyme maintains stable physicochemical properties and largely preserved catalytic activity under physiological conditions, supporting its suitability for long-term biomedical application in ROS-rich chronic wound environments.

### Fabrication and characterization of TA-Ce@PMCS MN

The fabrication process of the TA-Ce@PMCS MN is shown in Figure 3A. These MNs have transparent textures and notable flexibility, with a height of 500 µm and a base diameter of 200 µm (Figure 3B). As seen in Figure 3C, the tips displayed a porous structure, which is beneficial for the release of the TA-Ce nanozyme. Moreover, the TA-Ce nanoparticles were evenly distributed on the walls. The interaction between TA-Ce and PMCS was analyzed using rheology and XPS. As shown in Supplementary Figure S4, PMCS appeared in a liquid state (Gʹ ˂ G″), and the PMCS quickly formed a hydrogel when exposed to ultraviolet light for 5 s (Gʹ ˃G″). In contrast, the precursor solution of TA-Ce@PMCS started in a solid-like state (Gʹ ˃G″), and Gʹ increased rapidly with the irradiation of the subnet light. This was due to the formation of dynamic boronate ester bonds between the catechol groups on TA-Ce and the PBA on PMCS. Next, UV-induced carbon–carbon double bond polymerization further enhanced the cross-linking between molecules [45]. The XPS spectra of PMCS and TA-Ce@PMCS are shown in Supplementary Figure S5a; a distinct peak corresponding to Ce 3d elements at binding energies of 900 eV was present in the curves of TA-Ce@PMCS. The asymmetric B1s spectra of the TA-Ce@PMCS hydrogel in Figure 3D exhibited two deconvoluted peaks at 186.85 and 194.39 eV, which could be ascribed to the B–C/B–O in the phenylboronic group and B–O–C in the boronated esters group, respectively. However, only B1s peak in the PMCS curves at 186.85 eV is due to the B–C/B–O in the phenylboronic group (Supplementary Figure S5b). In addition, Ce 3d spectra of TA-Ce@PMCS could be deconvoluted into Ce^3+^ and Ce^4+^ (Supplementary Figure S5c), indicating TA-Ce was successfully wrapped in hydrogel and the chemical structure of TA-Ce remained unchanged after encapsulation. Mechanical performance serves as a critical indicator that determines whether MNs can successfully pierce the skin barrier. Related mechanical tests in Figure 3E reveal that increased TA-Ce loading amounts effectively enhance the stiffness and Young’s modulus of microneedle tips. Mechanical testing recorded compressive forces of 0.1–0.28 N for each microneedle when deformed to half of its tip height. This measured force far exceeds the 0.045 N threshold needed to pierce the human stratum corneum, as documented in previous studies [46, 47]. These findings confirm that TA-Ce@PMCS microneedles possess qualified mechanical rigidity for effective penetration of epidermal and subcutaneous tissues. SEM results demonstrate that TA-Ce@PMCS exhibits a more compact microstructure with reduced pore size compared to PMCS, further confirming increased crosslinking density and structural reinforcement (Supplementary Figure S6). Mechanistically, the enhanced mechanical strength can therefore be attributed to TA-Ce acting as a secondary physical–chemical crosslinking center, which increases network connectivity and improves resistance to deformation. In addition, the results of *in vivo* rat skin insertion experiments are shown in Supplementary Figure S7, and the microneedle arrays achieve effective penetration into the dermis with a depth of approximately ∼200 µm, indicating sufficient insertion capability to reach the epidermal–dermal interface and superficial dermis, which is adequate for diabetic wound microenvironment intervention and drug delivery.

Collectively, these results clarify that TA-Ce enhances hydrogel mechanical properties via additional crosslinking interactions and confirm that the microneedles possess appropriate tissue penetration depth for effective wound therapy.

Initially, biocompatibility is an essential property of biomaterials. The effect of TA-Ce@PMCS MN on the cells was assayed via trans-well plates. The MTT assay was used to quantitatively assess the toxicity of the materials (Figure 3F). There was no significant difference observed between TA-Ce@PMCS MN, PMCS MN, and control for L929 cells after coculture for 48 h, indicating that the MNs exhibited negligible toxicity. We further assessed cellular biocompatibility via live/dead fluorescent staining assays. After 1 day of cell incubation, the TA-Ce@PMCS microneedle group exhibited nearly identical green fluorescent signals compared with blank control samples (Figure 3G), which verified the favorable biosafety of this microneedle platform. Hyperglycemia, a diabetic wound, may produce excessive ROS because of its defense mechanisms. Excessive ROS can lead to local tissue damage and chronic inflammation [48]. Given the remarkable *in vitro* antioxidant capacity of TA-Ce nanozymes, we adopted DCFH-DA fluorescent probes to characterize their intracellular ROS elimination performance (Figure 3H). After H_2_O_2_ stimulation, Raw264.7 cells displayed obvious green fluorescence. Then, after treatment with MNs, green fluorescence almost disappeared in the TA-Ce@PMCS MN group and remained obvious green fluorescence in the PMCS MN group.

### ROS/glucose double-response of TA-Ce@PMCS MN

**Figure 4:**
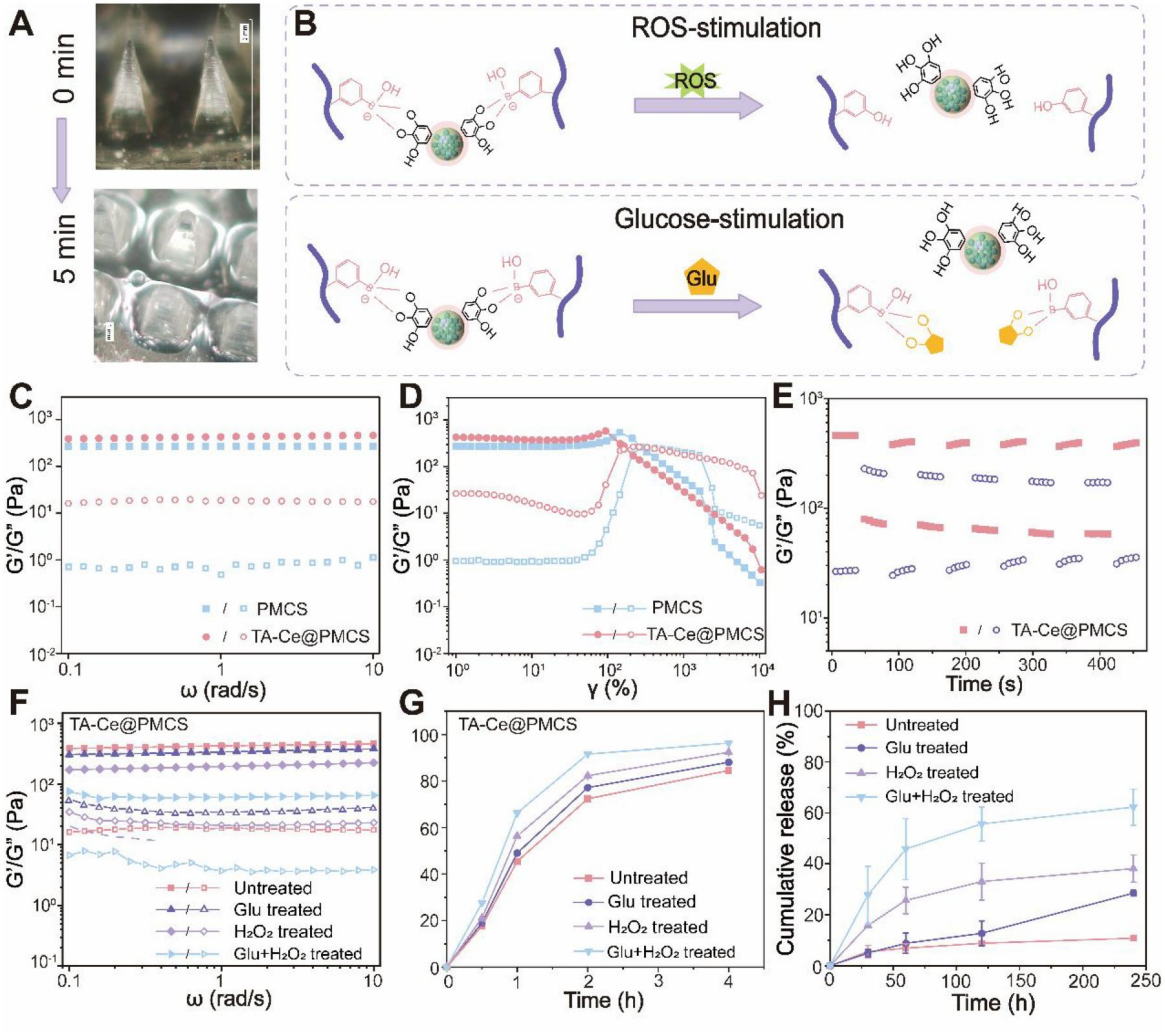
Characterization of the double-response properties. (**A**) The photograph of the TA-Ce@PMCS MN before and after immersion in the mixture of H_2_O_2_ and glucose. (**B**) Schematic illustration of the stimuli-responsive mechanism of the TA-Ce@PMCS MN. (**C**) G′ and G″ of TA-Ce@PMCS hydrogel as a function of different frequencies (*f* = 0.1–10 Hz) at fixed strain (γ = 1%), (**D**) amplitude strain (γ = 0.01–1000%) at fixed frequency (*f* = 1 Hz) and (**E**) alternate strains switching from 1 to 1000% for five cycles at fixed frequency (*f* = 1 Hz). (**F**) The rheological analysis of TA-Ce@PMCS hydrogel before and after H_2_O_2_ (1 mM) or glucose (25 mM) or dual stimuli conditions based on the frequency sweep (*f* = 0.1–10 Hz) test at a fixed strain (γ = 1%). (**G**) Swelling ratio of TA-Ce@PMCS hydrogel after different treatments. (**H**) Cumulative release of TA-Ce from TA-Ce@PMCS hydrogel after different treatments.

High blood glucose levels and excessive ROS are representative features of a diabetic wound [49]. Based on the intrinsic sensitivity of the boronated ester bond to glucose and ROS, the self-adaptive behaviors of TA-Ce@PMCS MN were examined. As shown in Figure 4A, after TA-Ce@PMCS MN was immersed in a H_2_O_2_ (1 mM) and glucose (25 mM) mixture solution for 5 min, the needle displays obvious swelling. The reason may be that phenyl borate ester bonds can be oxidized and degraded by H_2_O_2_ following the C–B bond breakage, and 1,2-diol structure in glucose substitutes the boronated ester bond upon the binding between TA-Ce and PMCS (Figure 4B) [50, 51]. Furthermore, the dynamic crosslink between TA-Ce and PMCS was tested via rheology (Figure 4C). The TA-Ce@PMCS hydrogel can consistently maintain a robust gel morphology with G′ higher than G″ in the frequency sweep test. The G′ of the TA-Ce@PMCS hydrogel could reach 410 Pa at 1 Hz and was higher than that of the PMCS hydrogel (270 Pa), indicating that TA-Ce nanoparticles could also enhance the elasticity property of the TA-Ce@PMCS hydrogel (Figure 4D). For the strain amplitude sweep assessment, the intersection point (G′ = G″) of the TA-Ce@PMCS hydrogel appeared at 180%, indicating the destruction of the hydrogel structure [52]. Moreover, a step-strain test was performed as shown in Figure 4E. Once the strain amplitude dropped back to 1%, both G′ and G′′ returned to their initial magnitudes, revealing fast gel reconstruction from the liquid state. The composite material retained the capacity to rebuild steady gel networks through five repeated loading-unloading cycles. This self-repairing behavior originates from reversible phenyl borate ester linkages dynamically formed between TA-Ce nanoparticles and PMCS polymer chains, as reported in relevant literature [53, 54]. Next, the ROS/glucose response of phenyl borate ester bonds was tested (Figure 4F). After being treated with glucose (25 mM) or H_2_O_2_ (1 mM), the G′ decreased from 405 to 318 kPa and 183 kPa, due to the responsive breakage of hydrogel networks. Notably, dual stimulation (glucose + H_2_O_2_) leads to the most pronounced reduction in G′ and G″ compared with single-stimulus conditions, indicating a significantly weakened hydrogel network. Specifically, boronate ester bonds are prone to being hydrolyzed under oxidative conditions, and boronate ester bonds could selectively coordinate with glucose to form stable boronate ester bonds when exposed to a high-glucose environment, making the TA-Ce@PMCS hydrogel system suitable for controlled and on-demand diabetic wound treatment.

Based on the sensitive ROS/glucose-induced structure adaptation property of the TA-Ce@PMCS hydrogel, the swelling and on-demand release behavior of TA-Ce were quantitatively analyzed. Figure 4G shows that the swelling ratio increased from 84.6 to 88.27% (glucose treatment), 92.66% (H_2_O_2_ treatment) and 96.37% (glucose + H_2_O_2_ treatment). Accordingly, the cumulative release of TA-Ce increased from 10.69 to 28.55% (glucose treatment), 38.28% (H_2_O_2_ treatment) and 62.39% (glucose + H_2_O_2_ treatment) for 240 h (Figure 4H). Specifically, the hydrogel’s ability to respond dynamically to the changing conditions within the wound microenvironment ensures precise and timely delivery of TA-Ce nanozyme to the target site for therapy, promoting effective and optimized regulation of the wound-healing process [55].

### TA-Ce@PMCS microneedles reprogram macrophage phenotype and metabolism via mitochondrial regulation

The inflammatory-oxidative stress cycle is a primary factor contributing to impaired healing in diabetic wounds. TA-Ce@PMCS demonstrates favorable antioxidant properties. While macrophages in the M1 phenotype effectively clear debris at the injury site, their release of proinflammatory mediators such as TNF-α, IL-1β and IL-6 can exacerbate tissue damage [56]. Therefore, the programmed transition from the proinflammatory M1 to the anti-inflammatory M2 phenotype during the inflammatory phase is crucial for promoting wound repair.

**Figure 5:**
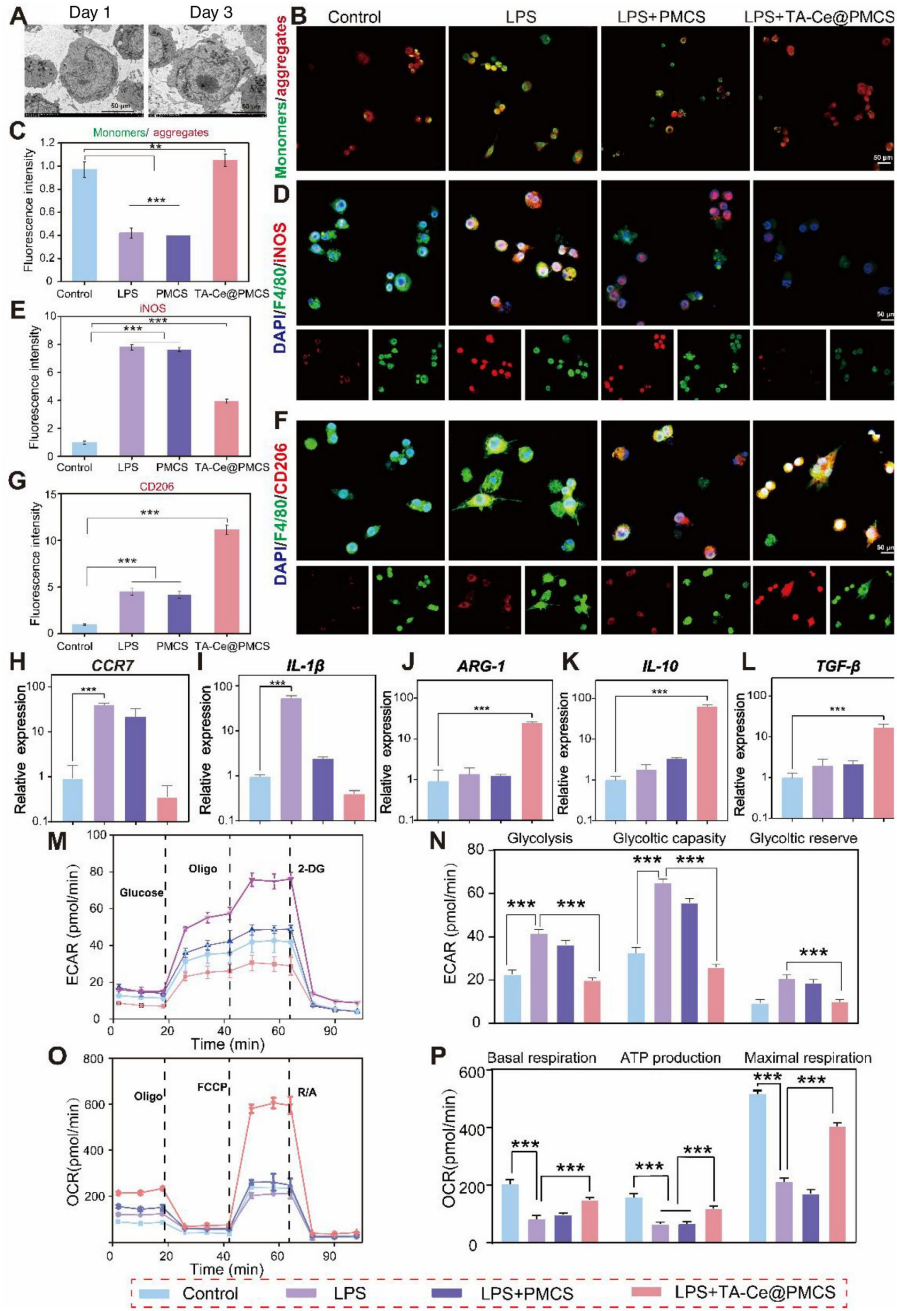
Macrophage phenotype regulation based on TA-Ce@PMCS MN. (**A**) The bio-TEM images of macrophages cultured with fluorescein-labeled TA-Ce@PMCS MN for 1 and 3 days. (**B**) Detection and (**C**) semiquantitative analysis of JC-1 mitochondrial MMP of macrophages after LPS stimulation. (**D**) Immunofluorescent images and (**E**) semiquantitative analysis of iNOS. (**F**) Immunofluorescent images and (**G**) semiquantitative analysis of CD206 in macrophages co-cultured with microneedle. The mRNA expression of M1 macrophage markers analysis of (**H**) CCR-7, (**I**) IL-1ɑ. The mRNA expression of M2 macrophage markers analysis of (**J**) ARG-1, (**K**) IL-10 and (**L**) TGF-β in RAW 264.7 cells after co-cultured with microneedle. (**M**) Real-time ECARs of macrophages in the glycolysis stress test and (**N**) semiquantitative analysis of glycolysis, glycolytic capacity and glycolytic reserve. (**O**) Real-time OCRs of macrophages in the cell mitochondrial stress test and (**P**) semiquantitative analysis of basal respiration, maximal respiration and ATP production. (*P < 0.05, **P < 0.01 and ***P < 0.001).

To explore the anti-inflammatory potential of the designed hydrogel material, co-culture experiments were conducted with RAW264.7 macrophage cells. Bio-TEM imaging clearly showed cellular uptake of nanoparticles. After 1 and 3 days of co-incubation, macrophages successfully engulfed these particles through typical endocytic pathways (Figure 5A). As shown in the fluorescence colocalization results (Supplementary Figure S8), TA-Ce nanozyme exhibits clear intracellular uptake in macrophages and shows strong overlap with mitochondrial signals, indicating efficient mitochondrial localization. The high degree of colocalization supports that TA-Ce can accumulate in mitochondria-rich regions, thereby enabling direct regulation of mitochondrial function. The endocytic uptake process exhibited an obvious time-varying trend, which demonstrates the continuous release of nanoparticles from the hydrogel matrix. It has been documented that macrophage polarization behavior is closely regulated by mitochondrial function and intracellular metabolic status [6]. In inflamed microenvironments, cellular bioenergetic balance is generally disrupted. Such metabolic disorders are mainly manifested by weakened respiratory complex activity, lowered MMP, impaired mitochondrial respiratory function, and limited adenosine triphosphate (ATP) synthesis efficiency.

Therefore, we investigated the effects of TA-Ce@PMCS MN microtargeting on mitochondrial integrity and function. JC-1 probes were used to detect MMPs in macrophages. LPS-stimulated macrophages showed increased monomeric JC-1 (green fluorescence), whereas co-culture with TA-Ce@PMCS reduced green fluorescence, indicating attenuation of mitochondrial depolarization (Figure 5B). Semi-quantitative analysis confirmed that TA-Ce@PMCS significantly restored MMPs in macrophages (Figure 5C), suggesting a protective role for the material on macrophage mitochondria under inflammatory stress.

To further validate the immunomodulatory capacity of TA-Ce@PMCS, macrophage polarization following co-culture was analyzed by immunofluorescence staining and qRT-PCR. M1 markers include CD86, TNF-α, inducible nitric oxide synthase (iNOS) and IL-1β, while M2 markers include arginase-1 (Arg-1), CD206 and IL-10. Immunofluorescence images showed that fluorescence intensity for the M1 marker iNOS gradually decreased, whereas that for the M2 marker CD206 increased in the TA-Ce@PMCS group compared with the control, LPS and PMCS groups (Figure 5D–G). qRT-PCR further confirmed that TA-Ce@PMCS MNs significantly downregulated proinflammatory cytokines (CCR7 and IL-1β) and upregulated anti-inflammatory mediators (Arg-1, TGF-β, and IL-10) in RAW264.7 macrophages (Figure 5H–L). These results demonstrate that TA-Ce@PMCS MNs concurrently alleviate oxidative stress and modulate inflammation, underscoring their therapeutic potential in diabetic wound healing. Extensive studies have established that macrophage immunometabolic reprogramming is tightly controlled by mitochondrial homeostasis, which is primarily regulated by the AMPK/SIRT1/PGC-1α signaling axis. Activation of AMPK promotes SIRT1-dependent deacetylation of PGC-1α, thereby enhancing mitochondrial biogenesis and OXPHOS, which is essential for M2 polarization [57, 58]. As shown in Supplementary Figure S9, the western blot results show that LPS stimulation markedly inhibits p-AMPK, SIRT1 and PGC-1α expression, indicating disrupted mitochondrial homeostasis and M1 polarization. In contrast, TA-Ce@PMCS MN significantly restores this signaling cascade, promoting mitochondrial recovery and M2 polarization. Importantly, pharmacological inhibition of AMPK phosphorylation using BAY-3827 (MCE) significantly attenuates downstream SIRT1/PGC-1α activation and reverses macrophage polarization effects, confirming the pathway dependency.

Serving as the core organelle for intracellular energy metabolism, mitochondria undergo functional alterations alongside metabolic state remodeling. Metabolic reprogramming in macrophages serves as a prerequisite for immune phenotypic transformation. Specifically, pro-inflammatory M1 macrophages rely on enhanced glycolysis and pentose phosphate pathway activity, alongside tricarboxylic acid cycle interruption and suppressed OXPHOS. In contrast, anti-inflammatory M2 macrophages maintain elevated OXPHOS and fatty acid oxidation levels to sustain their functional phenotype [15]. We adopted Seahorse extracellular flux detection to clarify if TA-Ce@PMCS reshapes macrophage polarization by tuning mitochondrial performance and subsequent cellular metabolism. This platform monitored metabolic shifts within inflamed macrophage populations treated with the composite microneedle material. Glycolysis stress tests tracked real-time extracellular acidification rates (ECARs) following successive administration of glucose, oligomycin and 2-deoxy-d-glucose, which allowed quantitative measurement of glycolysis-related indicators in macrophages (Figure 5M). Inflammatory macrophages induced by LPS displayed markedly elevated glycolysis levels, maximal glycolytic capacity and glycolytic reserve relative to the blank and pure PMCS groups. By contrast, macrophages cultured with TA-Ce@PMCS showed a notable drop in these glycolytic indexes (Figure 5N). Mitochondrial stress assays were further conducted to analyze cellular OXPHOS profiles. Real-time oxygen consumption rates (OCRs) were continuously recorded after the sequential addition of oligomycin, FCCP and rotenone, supporting the quantitative analysis of macrophage OXPHOS-related characteristics (Figure 5O). LPS stimulation obviously suppressed key OXPHOS indicators in macrophages. Both basal respiratory activity, ATP synthesis efficiency and maximal respiratory capacity were distinctly reduced in the inflammatory model group compared with untreated control cells (Figure 5P). Encouragingly, treatment with TA-Ce@PMCS effectively restored these impaired OXPHOS indexes in a recovering manner. After that, the *in vivo* release behavior and biosafety were evaluated by a subcutaneous implantation experiment. To trace the *in vivo* release of TA-Ce, the FITC-labeled TA-Ce nanoparticles were integrated with MNs for subcutaneous implantation in rats. As shown in Supplementary Figure S10a, a strong localized signal was observed at the implantation site on Day 1, followed by a gradual decrease over 4 and 7 days. In parallel, *ex vivo* fluorescence showed a corresponding time-dependent attenuation, indicating sustained release and progressive diffusion of TA-Ce from the microneedle matrix into surrounding tissues. Furthermore, to further evaluate systemic biosafety, major organs (heart, liver, spleen, lung, kidney and brain) were collected at Day 7 post-implantation for histological analysis. H&E staining results revealed no obvious pathological abnormalities, inflammatory infiltration or tissue damage in all examined organs, demonstrating the good *in vivo* biocompatibility and metabolic safety of the TA-Ce@PMCS microneedle system (Supplementary Figure S10b). Collectively, these additional data provide strong evidence for sustained local release behavior of TA-Ce *in vivo* and favorable systemic biosafety of the developed microneedle platform.

Collectively, these findings indicate that TA-Ce@PMCS reprograms inflammatory macrophages from glycolysis toward OXPHOS, facilitating a phenotypic shift from M1 to M2. The recovery of MMP and metabolic rewiring suggest improved mitochondrial function. Under inflammatory conditions, mitochondrial dysfunction lowers intracellular ATP and favors glycolysis. Beyond scavenging ROS, TA-Ce nanoparticles may also support cellular ATP homeostasis independently of mitochondria, thereby alleviating respiratory chain burden, creating a restorative buffer for mitochondrial recovery, and sustaining cellular energy supply.

### TA-Ce@PMCS MN accelerates diabetic full-thickness skin wound healing

**Figure 6:**
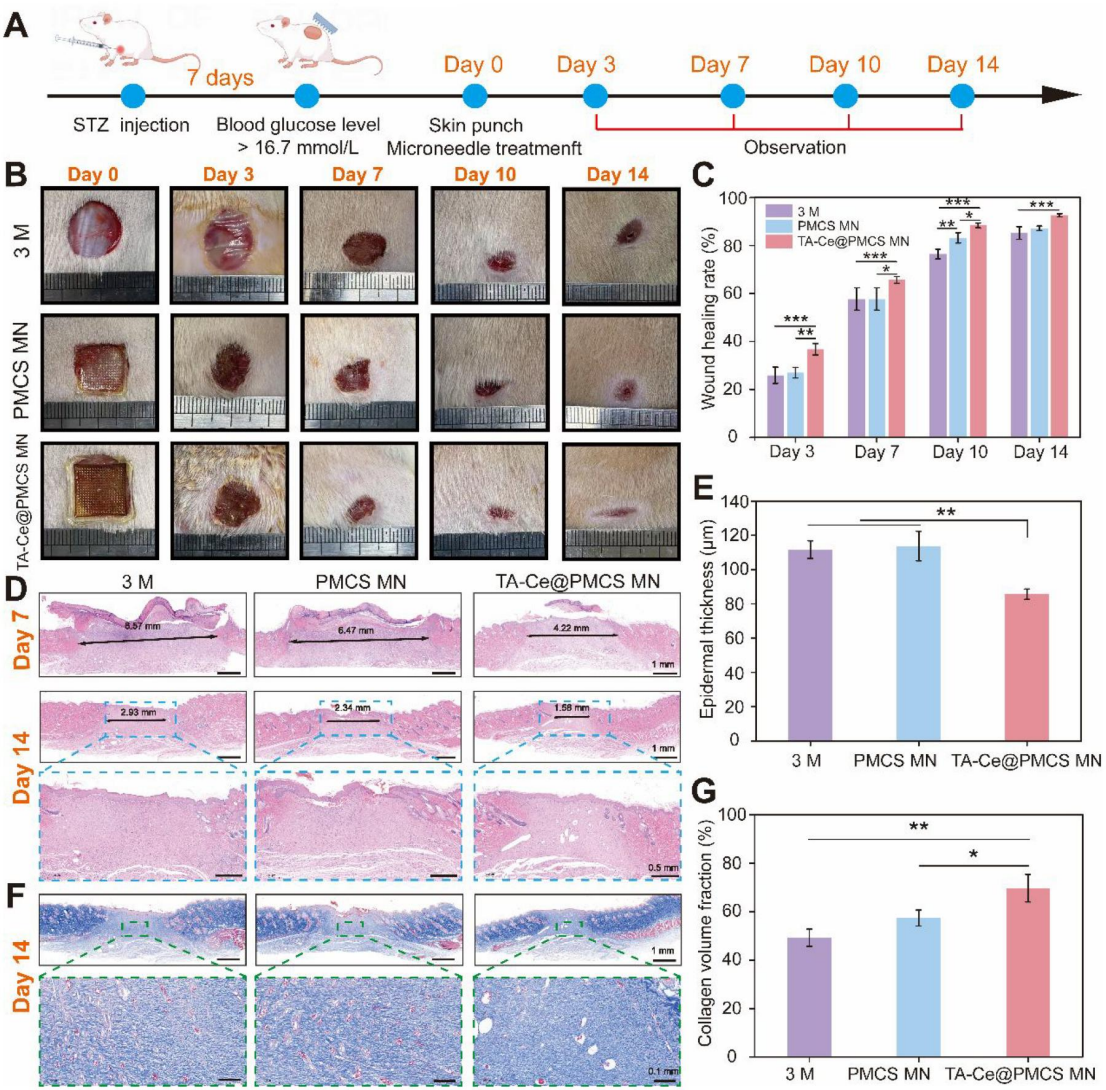
*In vivo* diabetic wound treatment efficacy of TA-Ce@PMCS MN. (**A**) A scheme showing the treatment schedule of the diabetic wound by different treatments. (**B**) Representative photographs of diabetic wounds after being treated with different samples for 0, 3, 7, 11 and 14 days. (**C**) Quantification analysis result of the wound healing rate during 14 days of treatment. (**D**) Representative images of H&E-stained wound tissues on the 7th and 14th day. (**E**) Quantitative analysis result of the epidermis thickness after 14 days of treatment. (**F**) Masson staining of the wound bed on Day 14. (**G**) The collagen volume fraction of the wound after 14 days of treatment. *n* = 3, *P < 0.05, **P < 0.01 and ***P < 0.001.

Diabetic skin lesions typically feature ROS overaccumulation, immune regulatory disorders and persistent inflammatory responses in wound tissues [59, 60]. Benefiting from superior free radical scavenging and anti-inflammatory bioactivities, the TA-Ce@PMCS microneedle system holds great potential for the treatment of diabetic cutaneous wounds. To further verify the *in vivo* therapeutic efficacy of this system, we constructed a classical diabetic rat model via streptozotocin injection, with standardized full-thickness skin wounds of 15-mm diameter created on the rat skin surface. A commercial wound dressing (Tegaderm™ film, 3M) served as the control (Figure 6A). Wounds were systematically monitored and photographed on Days 0, 3, 7, 10 and 14 post-treatments (Figure 6B and C). Notably, the TA-Ce@PMCS MN group exhibited a significantly reduced wound area after 3 and 7 days compared to both the 3M and PMCS MN groups. Interestingly, even on Day 14, substantial unhealed areas remained in the 3M and PMCS MN groups, with wound-healing rates of only 85.22 and 87.16%, respectively. In contrast, the TA-Ce@PMCS MN group showed markedly enhanced healing, achieving a wound closure rate of 92.64%.

We adopted two classic histological staining approaches, H&E and Masson’s trichrome staining, to comprehensively analyze the histological structure and regeneration quality of newly formed wound tissues. On Day 7, the TA-Ce@PMCS MN group displayed the smallest wound gap (4.22 mm), compared to 6.57 mm in the 3M group and 6.47 mm in the PMCS MN group. By Day 14, the TA-Ce@PMCS MN group exhibited complete re-epithelialization with a thin epidermis, regenerated blood vessels and newly formed hair follicles. In contrast, the 3M and PMCS MN groups showed undesirable thick epidermis and immature skin structures, indicating impaired healing (Figure 6D and E). Moreover, collagen deposition was significantly enhanced in the TA-Ce@PMCS MN group, with denser, better-aligned fibers and a higher collagen density (69.72%) than other groups (Figure 6F and G). These results demonstrate that TA-Ce@PMCS MN promotes wound healing by accelerating re-epithelialization, stimulating skin appendage regeneration, and enhancing collagen deposition.

**Figure 7:**
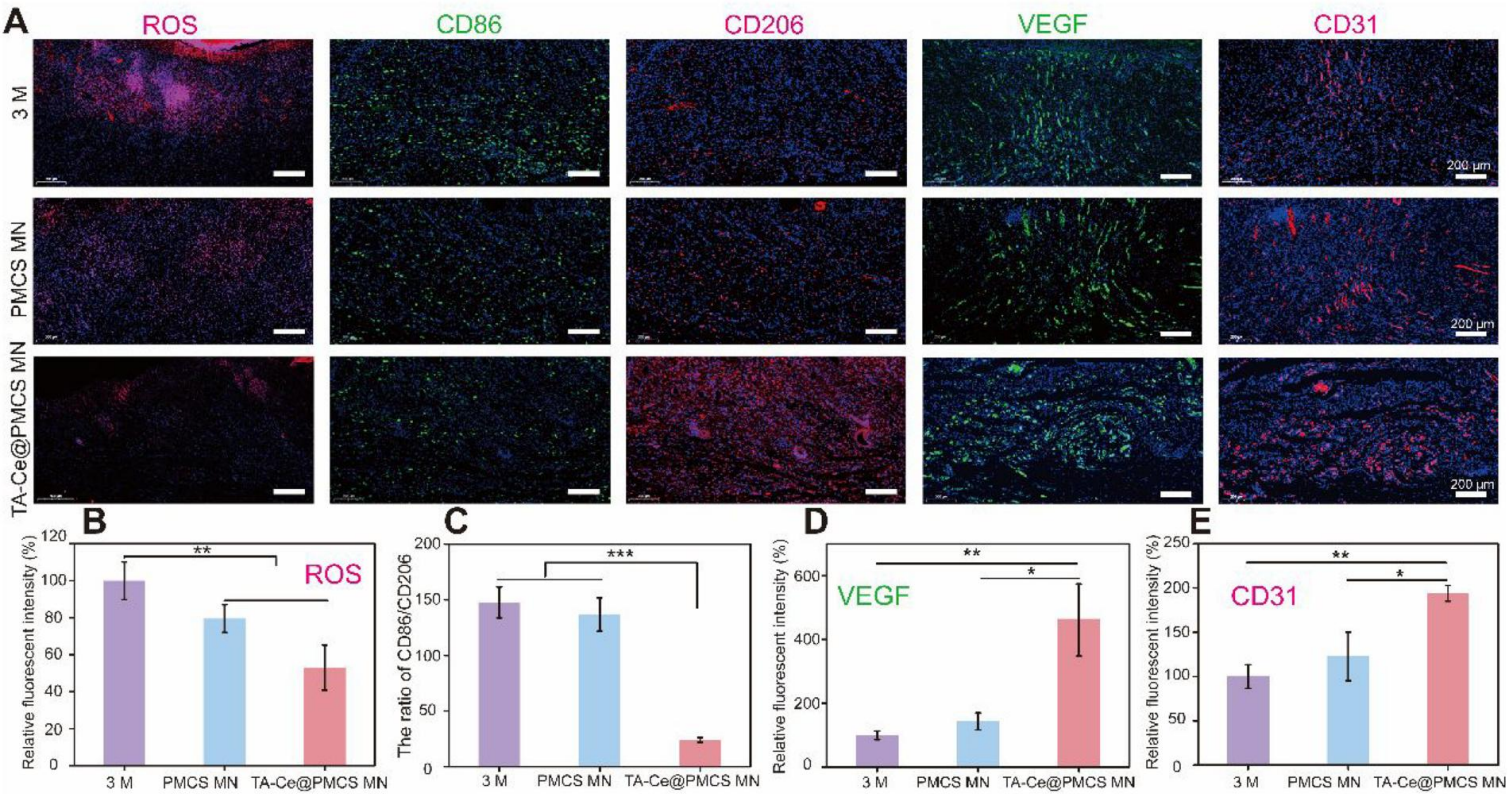
Assessment of *in vivo* antioxidant, anti-inflammatory and immunoregulatory ability of TA-Ce@PMCS MN. (**A**) Photographs of DHE immunofluorescence staining on the 7th day, CD86/CD206 on the 7th day, VEGF and CD31 on the 14th day. Quantitative analysis of (**B**) mean fluorescence intensity of ROS, the ratio of (**C**) CD86/CD206, (**D**) VEGF and (**E**) CD31. *n* = 3, *P < 0.05, **P < 0.01 and ***P < 0.001.

Motivated by the enhanced wound closure outcomes, we further investigated the healing mechanisms using immunofluorescence and histochemical staining. Chronic diabetic wounds are marked by prolonged inflammation, dominated by ROS accumulation and pro-inflammatory M1 macrophages [61]. Dihydroethidium staining on Day 7 revealed strong red fluorescence in the 3M and PMCS MN groups, indicating high ROS levels. In contrast, the TA-Ce@PMCS MN group showed markedly reduced fluorescence, confirming efficient ROS scavenging (Figure 7A and B). Additionally, phenotypic switching of macrophages is a critical step in inflammation resolution and tissue remodeling. Immunofluorescence analysis of M1 (CD86) and M2 (CD206) macrophages showed that the TA-Ce@PMCS MN group had the lowest CD86 and highest CD206 expressions. The CD86/CD206 ratio in the TA-Ce@PMCS MN group was only one-sixth of that in the 3M group, indicating a shift from pro-inflammatory M1 to pro-healing M2 macrophages (Figure 7A and C). This transition promoted the secretion of anti-inflammatory and angiogenic factors, thereby facilitating wound healing. Furthermore, enhanced neovascularization was observed via VEGF and CD31 staining. Treatment with TA-Ce@PMCS microneedles yielded notably denser neonatal vascular networks than all control groups (Figure 7A, D and E). Such enhanced vascular regeneration facilitates adequate oxygen delivery and nutrient transport at wound sites, which greatly promotes granulation tissue growth and accelerates the whole skin repair process.

In summary, the TA-Ce nanozyme enables microenvironment-responsive release within the wound bed, enhancing the anti-inflammatory and immunomodulatory functions of TA-Ce@PMCS MN *in vivo*. This leads to mitigated inflammation, remodeling of the diabetic wound microenvironment, promotion of tissue regeneration, and accelerated wound healing.

## Conclusion

In this study, we have developed a microenvironment-responsive metal-phenolic nanozyme-based MN, TA-Ce@PMCS MN, for programmed regulation of diabetic wound regeneration. By integrating TA-Ce nanozymes into a PBA-functionalized CS hydrogel, we engineered a smart system capable of sensing and adapting to the pathological cues of diabetic wounds-specifically, elevated ROS and glucose levels, through breakable boronated ester bonds. The TA-Ce nanozyme not only served as a dynamic crosslinker but also conferred potent dual SOD- and CAT-mimetic activities, enabling efficient scavenging of multiple reactive oxygen and nitrogen species. This effectively disrupts the self-sustaining ROS-inflammation cycle characteristic of diabetic impairments. In a diabetic rat full-thickness wound model, TA-Ce@PMCS MN significantly accelerated wound closure, enhanced re-epithelialization, stimulated collagen deposition and fostered functional neovascularization. The responsive release behavior ensures spatiotemporally precise delivery of therapeutic nanozymes deep into the wound bed, aligning treatment kinetics with the dynamic healing process. The integration of multifunctional MPNs with microneedle technology offers a powerful and versatile platform for the treatment of chronic wounds and other inflammatory tissue disorders.

## Supplementary Material

rbag153_Supplementary_Data

## Data Availability

The data that support the findings of this study are available from the corresponding author upon reasonable request.
