# Supplementary material for: Glucose/ROS dual-responsive mitochondria-targeting nanozyme microneedles remodel mitochondrial homeostasis to orchestrate immune metabolic reprogramming for diabetic foot ulcer healing
Source: Regen Biomater. 2026 Jul 21;13:rbag153. doi: 10.1093/rb/rbag153 (PMC13457093; doi:10.1093/rb/rbag153)
Supplement: Supplementary file 1 [file rbag153_supplementary_data.docx]

Supporting Information

**Glucose/ROS Dual-Responsive Mitochondria-Targeting Nanozyme Microneedles Remodel Mitochondrial Homeostasis to Orchestrate Immune Metabolic Reprogramming for Diabetic Foot Ulcer Healing**

*Qi Dong ^a, †^, Siyu Xiong ^a, †^, Bing Shu ^c, †^,* *Zhiwei Cai ^a^, Shangqing Wang ^a^,*

*Yang Ming ^d^, Xiongwei Dong ^a, *^, Peng Chen ^b, *^,* *Zhaowei Zhang ^a, *^*

^a^ School of Bioengineering and Health, Wuhan Textile University, Wuhan 430073, PR China

^b^ Department of Pharmacy, Renmin Hospital of Wuhan University, Wuhan 430060, PR China

^c^ Department of Neurosurgery, Zhongnan Hospital of Wuhan University, Wuhan 430060, PR China

^d^ Hubei Integrative Technology and Innovation Center for Advanced Fiberous Materials, Wuhan 430073, PR China

E-mail addresses: xwdong@wtu.edu.cn (X. Dong), [2282968908@qq.com (P. Chen),](mailto:18243398380@163.com%20(X.%20Sun),) zwzhang@whu.edu.cn (Z. Zhang)

^†^ These authors contributed equally to this work.

**Figure S1.** Energy-dispersive spectroscopy (EDS) confirms the elemental composition of Ce-TA nanoparticles.

**Table S1.** The inset table quantifies atomic and mass fractions, indicating cerium (Ce) content of 2.75% (atomic fraction) and 23.33% (mass fraction), confirming efficient cerium incorporation.

| Element | Atomic Fraction (%) | Mass Fraction (%) |
| --- | --- | --- |
| C | 72.55 | 52.75 |
| O | 24.70 | 23.93 |
| Ce | 2.75 | 23.33 |

**Figure S2.** High-resolution C 1s XPS spectra identify characteristic peaks of aromatic groups in tannic acid.

Figure S3. long-term stability of TA-Ce nanozyme after immersing in PBS for 14 days. (a) The size. (b) DPPH radical scavenging activity. **p < 0.05, **p < 0.01, ***p < 0.001*.

**Figure S4.** The gel time of the hydrogel.

**Figure S5.** (a) XPS survey spectrum of PMCS and TA-Ce@PMCS. (b) B 1s XPS spectra of PMCS. (c) High-resolution XPS patterns of Ce 3d peaks in TA-Ce@PMCS.

Figure S6. Mechanical reinforcement mechanism and in vivo penetration performance of TA-Ce@PMCS hydrogel microneedles.

Figure S7. In vivo skin penetration performance of TA-Ce@PMCS hydrogel microneedles. Representative gross image of microneedle application on rat skin and corresponding H&E-stained histological section showing effective penetration into the skin tissue.

Figure S8. Mitochondrial colocalization of TA-Ce in macrophages. Mitochondria were stained with MitoTracker (red), TA-Ce was labeled with intrinsic/fluorescent signal (green), and nuclei were counterstained with DAPI (blue).

Figure S9. Activation of the AMPK/SIRT1/PGC-1α signaling pathway in macrophages is regulated by TA-Ce@PMCS MN. (a) Representative Western blot analysis of PGC-1α, SIRT1, phosphorylated AMPK (p-AMPK), total AMPK, and β-actin in RAW264.7 macrophages under different treatments (sham, vehicle, LPS, TA-Ce@PMCS, and AMPK inhibitor BAY-3827). (b–d) Densitometric quantification of PGC-1α, SIRT1, and p-AMPK protein expression normalized to β-actin or total AMPK, respectively. Data are presented as mean ± SD (n = 3). **p < 0.05, **p < 0.01.*

Figure S10. In vivo release behavior and biosafety evaluation of TA-Ce@PMCS microneedles. (a) In vivo fluorescence imaging of FITC-labeled TA-Ce@PMCS microneedles at 1, 4, and 7 days. (b) Representative H&E staining of major organs (lung, heart, spleen, liver, kidney, and brain) collected at day 7 post-treatment.

**Table S2**. Primers used in the RT-qPCR assay

| Transcription | Prime sequences |
| --- | --- |
| CCR7 | Forward: CAGGTGTGCTTCTGCCAAGAT |
|  | Reverse: GGTAGGTATCCGTCATGGTCT |
| IL-1β | Forward: CACTACAGGCTCCGAGATGAACAAC |
|  | Reverse: TGTCGTTGCTTGGTTCTCCTTGTAC |
| IL-6 | Forward: CTGCAAGAGACTTCCATCCAG |
|  | Reverse: AGTGGTATAGACAGGTCTGTTGG |
| TNF-α | Forward: ATGTCTCAGCCTCTTCTCATTC |
|  | Reverse: GCTTGTCACTCGAATTTTGAGA |
| ARG1 | Forward: CTCCAAGCCAAAGTCCTTAGAG |
|  | Reverse: GGAGCTGTCATTAGGGACATCA |
| CD163 | Forward: GGTGGACACAGAATGGTTCTTC |
|  | Reverse: CCAGGAGCGTTAGTGACAGC |
| IL-10 | Forward: CTTACTGACTGGCATGAGGATCA |
|  | Reverse: GCAGCTCTAGGAGCATGTGG |
| TGF-β | Forward: CCAGATCCTGTCCAAACTAAGG |
|  | Reverse: CTCTTTAGCATAGTAGTCCGCT |
